# Comments on differences in classification of the superfamily Noctuoidea (Insecta, Lepidoptera) between Eurasia and North America

**DOI:** 10.3897/zookeys.264.4441

**Published:** 2013-02-06

**Authors:** J. Donald Lafontaine, B. Christian Schmidt

**Affiliations:** 1 Canadian National Collection of Insects, Arachnids, and Nematodes, Biodiversity Program, Agriculture and Agri-Food Canada, K.W. Neatby Bldg., 960 Carling Ave., Ottawa, Ontario, Canada K1A 0C6; 2 Canadian Food Inspection Agency, Canadian National Collection of Insects, Arachnids, and Nematodes, K.W. Neatby Bldg., 960 Carling Ave., Ottawa, Ontario, Canada K1A 0C6

**Keywords:** North America, Europe, Ophiusini, Poaphilini, Dilobinae, Raphiinae, Amphipyrinae, Psaphidinae, Oncocnemidini, Athetiina, Xyleninae, Hadeninae, Noctuinae, *Arctia*, *Pararctia*, *Platarctia*, Lymantriinae

## Abstract

An attempt is made to bring consensus between the classifications of the Noctuoidea in Europe and North America. Twelve points of disagreement between the check lists from the two regions are discussed and solutions recommended.

## Introduction

There has been excellent collaboration among noctuoid specialists in Europe and North America over the past 30 years, highlighted by the collaboration between Michael Fibiger (Denmark) and Don Lafontaine (Canada) in bringing the noctuoid classification used in Europe and North America into harmony. Michael’s death on 16 February 2011 was a tragic loss for his family, friends, and the Lepidoptera community. Michael’s main research focus for the last 22 years of his life was his initiation, production, and coordination of a massive 13 volume series of books on the noctuid fauna of Europe. The final volume completing the series was finished and published by his colleagues in 2011. This volume included a check list of the four quadrifid families of Noctuoidea (i.e., Noctuidae, sensu lato) of Europe (Fibiger et al. 2011), based on the 13 published volumes, recent phylogenetic work by Zahiri et al. (2011, 2012), and the North American check list (Lafontaine and Schmidt 2010). The 2011 European list departs from the North American list in 12 taxonomic areas. They are discussed below in the taxonomic sequence of Lafontaine and Schmidt (2010).

## Results

**1. **The phylogeny and classification within the subfamily Lymantriinae is still very fragmentary, and attempts at systematic overviews have been limited to regional accounts. Ferguson (1978) recognized two tribes (Orgyini and Lymantriini) within a single subfamily (Lymantriinae) for North American taxa of the Lymantriidae, allowing that African and Asian genera would likely represent additional subfamilies. Under Ferguson’s definition, the composition of the subfamily and family were the same, there being only a single subfamily in the family Lymantriidae. Kitching and Rawlins [1998] did not present any subdivisions within the Lymantriidae. Holloway (1999) maintained Ferguson’s concept of one subfamily divided into tribes, and recognized three additional tribes based on Indo-Australian genera. Benkhelil (1999) raised Orgyiini and Lymantriini to subfamilies and discussed synapomorphies between European genera and those studied by Ferguson (1978), while transferring *Euproctis* Hübner to Orgyiinae from Lymantriinae. The Indo-Australian genera and tribes proposed by Holloway (1999) were not included by Benkhelil (1999). Most recently, Witt and Ronkay (2011) reconcile existing classifications by arranging the Erebidae: Lymantriinae into two tribes, corresponding to Benkhelil’s (1999) concepts of Orgyiinae and Lymantriinae, and further dividing each tribe into subtribes based on the five tribal groups of Holloway (1999): Lymantriini with Lymantriina, Arcornithina, Leucomina, and Orgyiini with Orgyiina and Nygmiina. This classification would result in changes of tribal and subtribal placement for all genera occurring in North America. However, we note that the current concept of Orgyiini (Benkhelil 1999, Witt and Ronkay 2011) appears not to be monophyletic, since molecular data (Mitchell et al. 2006) indicate that *Euproctis* (Orgyiini: Nygmiina) is more closely related to *Lymantria* Hübner (Lymantriini: Lymantriina) than to *Dasychira* Hübner or *Orgyia* Ochsenheimer (Orgyini: Orgyina). Until a more comprehensive systematic study of the Lymantriinae is undertaken and suprageneric taxa are put into a phylogenetic hierarchy, we follow Holloway (1999) and retain the family-group taxa within the Lymantriinae as tribes (Lafontaine and Schmidt 2010, 2011).

**2.** Witt et al. (2011) synonymized *Platarctia* Packard and *Pararctia* Sotavalta under *Arctia* Schrank. Although a very broad concept of *Arctia* could be justified by including a number of traditionally recognized arctiine genera, the current concept of *Arctia* as defined by Witt et al. renders *Arctia* polyphyletic as neither of these two genera are sister groups to *Arctia* (Schmidt 2007, Dubatolov 2008). We therefore maintain *Pararctia* and *Platarctia* as valid genera.

**3.** The Boletobiinae, Aventiinae, Eublemminae, and Phytometrinae are treated as subfamilies in Lafontaine and Schmidt (2010), but as tribes of the Boletobiinae by Fibiger et al. (2011). They are downgraded to tribes in Lafontaine and Schmidt (2012) on the basis of the results in Zahiri et al. (2012), resulting in agreement between the two lists.

**4.** The genus *Colobochyla* Hübner is included in the subfamily Hypeninae in Lafontaine and Schmidt (2010), but in the subfamily Boletobiinae, tribe Phytometrini in Fibiger et al. (2011). The genus was included in the Phytometrinae by Fibiger and Lafontaine (2005) and Beck (1999–2000) in order to restrict the Hypeninae to the genus *Hypena* Schrank, because of its many peculiarities, such as the appendiculate tooth on the larval crochets. The adult and larva are similar in habitus to those of species of *Phytometra* Haworth, but there are no definitive derived characters that associate *Colobochyla* with either the Hypeninae or the Phytometrinae. The genus was included in the Hypeninae because DNA results in Zahiri et al. (2011) indicated a sister-group relationship between *Hypena* and *Colobochyla* (Bootstrap support 68%, Bremer support value 13 [68/13]). Support for the subfamily Boletobiinae, which now includes the Phytometrini as a tribe, is 98/13, suggesting *Colobochyla* is not a phytometrine. More recently, expanded results from Zahiri et al. (2012) place *Colobochyla* as the sister group to the Hypeninae without significant support, but the support for the Boletobiinae is 100/5, clearly excluding *Colobochyla* from the Boletobiinae and Phytometrini. So, until evidence for a better phylogenetic placement for *Colobochyla* is brought forward, we retain it in the Hypeninae.

**5.** In North America the genera *Achaea* Hübner, *Allotria* Hübner, *Argyrostrotis* Hübner, *Cutina* Walker, *Gondysia* Berio, *Mimophisma* Hampson, *Ophisma* Guenée, and *Parallelia* Hübner are now included in the Erebidae, Erebinae, Poaphilini, the tribal name based on a synonym of *Argyrostrotis*. Three of these genera (*Achaea*, *Mimophisma*, and *Ophisma*) are transferred into the Poaphilini here on the basis of the molecular results of Zahiri et al. (2012). This tribe has not been recognized in Europe, but Goater et al. (2003) treated it as “the *Parallelia* genus-group” of the subtribe Ophiusina, and gave a list of characters that define the group, the most obvious of which is the eversible coremata ballooning out from the outer proximal surface of the valve in most genera. They included the European genera *Grammodes* Guenée and *Dysgonia* Hübner in the group, as well as the Asian genera *Achaea*, *Bastilla* Swinhoe, *Buzara* Walker, *Euphiusa* Hampson, *Ophisma*, and *Parallelia*. Holloway (2005) also arranged the genera of the Ophiusini into two generic groupings, one being the “*Achaea/Parallelia* complex,” which includes many of the same genera as Goater et al., but adds *Chalciope* Hübner, *Macaldenia* Moore, *Pindara* Moore, and provisionally *Oxyodes* Guenée. This segregate of the Ophiusini is recognized as the tribe Poaphilini and sister to the Ophiusini with bootstrap/Bremer support values in Zahiri et al. (2012) of 100/19 and 100/16 respectively and associated as sister taxa with 93/6 support values. As a result, we transfer the European genera *Grammodes* and *Dysgonia* from the Ophiusini to the Poaphilini.

**6.** The subfamily Raphiinae was synonymized with the Dilobinae by Fibiger et al. (2009) and followed by Lafontaine and Schmidt (2010) in the North American list. The two subfamilies were separated again by Yela and Zahiri (2011) and Fibiger et al. (2011), mainly on larval autapomorphies of each of the two genera comprising these subfamilies. The problem of determining the systematic position of Dilobinae has been that the single constituent species (*Diloba caeruleocephala* (Linnaeus)) exhibits many autapomorphic traits that obscure its relationship to the Raphiinae / Pantheinae group, the most likely closest relatives (Miller 1991, Zahiri et al. 2012). Fibiger et al. (2009) united *Diloba* and *Raphia* on presumed unique synapomorphies in the genitalic structure, but an examination of a broader sampling of global pantheine genera (BCS, unpubl. data) shows these characters to also be present in the Pantheinae. To date, molecular results provide no support for Dilobinae as the sister group to Raphiinae (Mitchell et al. 2006, Zahiri et al. 2012). We therefore follow Yela and Zahiri (2011) and Fibiger et al. (2011) in treating the three taxa, Dilobinae, Raphiinae, and Pantheinae, as separate subfamilies.

**7.** The European list treats the Amphipyrinae and Psaphidinae as subfamilies with the Feraliini a tribe of the latter following Poole (1995), Fibiger and Lafontaine (2005), and Fibiger and Hacker (2007). In the North American list the subfamily Amphipyrinae includes three tribes, Amphipyrini, Psaphidini (with four subtribes) and Stiriini (with four subtribes). The Amphipyrini is essentially based on the genus *Amphipyra* Ochsenheimer and characterized by its many peculiarities. Many characters form a mosaic in distribution, such as the uniordinal larval crochets in Amphipyrini and Feraliina and biordinal crochets in the Psaphidina and Cuculliinae. Molecular work by Mitchell (2006, Fig. 4) suggests a sister group relationship between the Amphipyrinae and Psaphidini with the Stiriini being the sister group to them. The close relationship between Psaphidini and Amphipyrini is also highlighted by the recent discovery of species exhibiting larval and adult characters clearly associating these lineages (Wagner et al. 2008). Until more molecular work has been done to bring better resolution to the phylogenetic relationships of the tribes and subtribes of the clade, we feel it is better to treat the whole clade as a single subfamily – the Amphipyrinae.

**8.** In Europe the situation with *Sympistis* Hübner and *Oncocnemis* Lederer seems relatively simple with four species of *Sympistis* being diurnal arctic-alpine species and five species of *Oncocnemis* being nocturnal and desert loving. In North America, however, *Sympistis* is very large (176 species) and structurally complex and the four *Sympistis* s.s. species are not only nested within the *Sympistis*/*Oncocnemis* complex as a whole, but one species (*Sympistis funebris* Hübner) does not form a monophyletic clade with the other diurnal species that formerly constituted *Sympistis*. *Sympistis funebris* is the sister species to a clade of species formerly considered a separate genus (*Apharetra* Grote), so *Sympistis* s.s. is polyphyletic. The genera of the Oncocnemidini were revised by Troubridge (2008), who treated many former genera as species groups within an expanded concept of *Sympistis*. We follow Troubridge in treating *Oncocnemis* as a synonym of *Sympistis* and including the four species of the former *Sympistis* (in two different species groups).

**9.** *Sympistis nigrita* (Boisduval), described from the Alps, was treated as a Holarctic species by Ronkay and Ronkay (1995) and Troubridge (2008) by virtue of considering the northern Holarctic taxon *Sympistis zetterstedtii* (Staudinger) as a subspecies of *Sympistis nigrita*. *Sympistis zetterstedtii*, stat. rev., differs from *Sympistis nigrita* in that the fields of cornuti in the male vesica are concentrated into two dense patches, not scattered over the apical half of the vesica as in *Sympistis nigrita*, and the subbasal diverticulum of the vesica in *Sympistis zetterstedtii* is minute, not pouch-like as in *Sympistis nigrita*. In the female genitalia of *Sympistis zetterstedtii* the ductus bursae enters the corpus bursae on the side of the near the posterior end, whereas it enters at the posterior end of the corpus bursae in *Sympistis nigrita*. The barcodes of the two species are 2.8% different (Mutanen et al. 2012). Populations of *Sympistis zetterstedtii* in Yukon and Alaska have dark hindwings, like those from Fennoscandia, however, their barcodes are the same as those from Greenland and the barcodes of North American populations differ from those from northern Europe by more than 1%, so we treat the North American populations as *Sympistis zetterstedtii* ssp. *kolthoffi* (Aurivillius, 1890). We see no structural differences among populations of *Sympistis zetterstedtii*, unlike the situation between *Sympistis zetterstedtii* and *Sympistis nigrita*. As a result of these data, we treat *Sympistis nigrita* as being endemic to the Alps and *Sympistis zetterstedtii* as a northern Holarctic species.

**10.** *Protoschinia scutosa* ([D. & S.]) is represented in North America by a separate species, *Protoschinia nuchalis* (Grote), which is currently included in the genus *Schinia* Hübner on the basis of synonymy of the genera by Matthews (1991). The status of the two genera will be addressed in an upcoming revision by Michael Pogue, but in the interim we follow the European lead in returning *nuchalis* to *Protoschinia* Hardwick, mainly because the barcodes suggest that *Protoschinia* is more closely related to *Heliothis* Ochsenheimer and *Helicoverpa* Hardwick than it is to *Schinia*.

**11. **The European check list follows Fibiger and Lafontaine (2005), Fibiger and Hacker (2005) and Lafontaine and Fibiger (2006) in arranging the genera in Noctuinae s.l. of Poole (1995) into three subfamilies, Xyleninae, Hadeninae, and Noctuinae s.s. However, Fibiger and Lafontaine (2005) stated that the Xyleninae cannot be defined on any shared derived character states and is probably a paraphyletic group with respect to some tribes in the Hadeninae. Similarly, Yela and Zahiri (2011) stated there are no derived character states to support the Xyleninae. These results are not surprising because the molecular work of Mitchell et al. (2006) shows that the tribes Pseudeustrotiini, Phosphilini, Prodeniini, Elaphriini, Caradrinini, Dypterygiini, and Actinotiini are external to the [[Apameini + Xylenini] + Hadeninae] + Noctuinae clade, and it appears that the Hadeninae do not form a monophyletic group, with some tribes more closely related to tribes in the Xylenini. The result is that either these tribes need to be combined as tribes of a single subfamily, the Noctuinae s.l., or they need to be arranged in 10 to 20 poorly-defined subfamilies, in order to retain monophyletic taxa. The monophyly of the subfamily Noctuinae s.l. is very well supported by the molecular results of Mitchell et al. (2006), and by morphology (clasper located in middle of valve, larva with dorsally-grooved spinneret), and this clade contains the true cutworms, many of which are significant agricultural pests. As a result, we believe the best option is to arrange the tribes in a single expanded concept of the Noctuinae as was done by Lafontaine and Schmidt (2010) for the North American taxa.

**12.** The subtribe Athetiina was originally constructed by Fibiger and Lafontaine (2005) by dropping the ‘*is*’ ending from *Athetis* Hübner to create the subtribal name Athetina. Later, when it was revealed that the name was a homonym of the Athetina in Coleoptera, Staphylinidae, based on *Atheta* Thomson, the stem was changed by dropping only the ‘*s*’ to create Athetiina to avoid homonomy.

The subtribe includes several hundred species, mainly Old World, which can be arranged in three distinctive groups: *Athetis* Hübner, with parallel-sided valves, a clasper complex positioned near the apex of the valve on (or near) the ventral margin, and the sacculus is very long; *Hydrillula* Tams is mainly an African group with parallel-sided valves, apically expanded and rounded at the cucullus, and the clasper is spine-like; *Proxenus* Herrich-Schäffer, mainly eastern Asian and North American, valves greatly expanded from a small base to a large rounded apex with the clasper in the middle of the apical area, the sacculus is very small. *Athetis* and *Proxenus* occur in North America and were treated as genera of the Athetiina by Fibiger and Lafontaine (2005), Fibiger and Hacker (2005), and Lafontaine and Schmidt (2010). The three genera were treated as subgenera of *Athetis* by Fibiger and Hacker (2007) and Fibiger et al. (2011). The loss of the uncus makes an easy and reliable character to define this group, and is the main justification for the single genus *Athetis*; however, this same character was the main basis for the subtribal grouping to associate these three genera as a monophyletic group. The structural differences among the three groups are consistent and significant, so it seems pointless to define the genus *Athetis* and Athetiina on the same characters and have a subtribe with only one genus. We therefore treat *Athetis* and *Proxenus* as valid genera, not as subgenera of *Athetis*.

## Discussion

The purpose of a classification, and resulting check lists, are to organize, store, and communicate information about organisms and their names. In the not so distant past, different classifications were frequently in use on different continents, and even among countries and regions, severely hampering effective communication and transfer of biological information. Consensus in classification is becoming an increasingly important issue with globalization of data-bases and information available on the World Wide Web. There has been a huge amount of progress in the past 20 years in the development of a consensus classification of the superfamily Noctuoidea between North America and Eurasia because of cooperation and collaboration of researchers. Classifications are not static, but will continue to change and adapt as new data and new ways of interpreting data are brought forward. We believe that transparency, communication, and collaboration will aid in the process of maintaining stability while continuing to change. We hope that this contribution will be a step forward in continuing this process.
